# Accelerating thermokarst lake changes on the Qinghai–Tibetan Plateau

**DOI:** 10.1038/s41598-024-52558-7

**Published:** 2024-02-05

**Authors:** Guanghao Zhou, Wenhui Liu, Changwei Xie, Xianteng Song, Qi Zhang, Qingpeng Li, Guangyue Liu, Qing Li, Bingnan Luo

**Affiliations:** 1 Department of Geological Engineering, Qinghai University, Xining, 810016 Qinghai China; 2 Cryosphere Research Station on the Qinghai-Tibet Plateau, State Key Laboratory of Cry-osphere Sciences, Northwest Institute of Eco-Environment and Resources, Chinese Academy of Sciences, Lanzhou, 730000 China; 3 Xining Center for Integrated Natural Resources Survey, China Geological Survey, Xining, 810000 Qinghai China

**Keywords:** Cryospheric science, Climate-change impacts

## Abstract

As significant evidence of ice-rich permafrost degradation due to climate warming, thermokarst lake was developing and undergoing substantial changes. Thermokarst lake was an essential ecosystem component, which significantly impacted the global carbon cycle, hydrology process and the stability of the Qinghai–Tibet Engineering Corridor. In this paper, based on Sentinel-2 (2021) and Landsat (1988–2020) images, thermokarst lakes within a 5000 m range along both sides of Qinghai–Tibet Highway were extracted to analyse the spatio-temporal variations. The results showed that the number and area of thermokarst lake in 2021 were 3965 and 4038.6 ha (1 ha = 10,000 m^2^), with an average size of 1.0186 ha. Small thermokarst lakes ($<\,$1 ha) accounted for 85.65% of the entire lake count, and large thermokarst lakes ($>\,$10 ha) occupied for 44.92% of the whole lake area. In all sub-regions, the number of small lake far exceeds 75% of the total lake number in each sub-region. R1 sub-region (around Wudaoliang region) had the maximum number density of thermokarst lakes with 0.0071, and R6 sub-region (around Anduo region) had the minimum number density with 0.0032. Thermokarst lakes were mainly distributed within elevation range of 4300 m–5000 m a.s.l. (94.27% and 97.13% of the total number and size), on flat terrain with slopes less than 3^∘^ (99.17% and 98.47% of the total number and surface) and in the north, south, and southeast aspects (51.98% and 50.00% of the total number and area). Thermokarst lakes were significantly developed in warm permafrost region with mean annual ground temperature (MAGT) > − 1.5 ^∘^C, accounting for 47.39% and 54.38% of the total count and coverage, respectively. From 1988 to 2020, in spite of shrinkage or even drain of small portion of thermokarst lake, there was a general expansion trend of thermokarst lake with increase in number of 195 (8.58%) and area of 1160.19 ha (41.36%), which decreased during 1988–1995 (− 702 each year and − 706.27 ha/yr) and then increased during 1995–2020 (184.96–702 each year and 360.82 ha/yr). This significant expansion was attributed to ground ice melting as rising air temperature at a rate of 0.03–0.04 ^∘^C/yr. Followed by the increasing precipitation (1.76–3.07 mm/yr) that accelerated the injection of water into lake.

## Introduction

The Qinghai–Tibet Plateau is the highest and most widely distributed plateau permafrost on Earth, which is one of the most sensitive areas to climate change. The permafrost area is about 1.06 $\times$ 10^6^ km^2^^1^, and the ground ice volume in permafrost is 12,700 km^3^^2^. Due to climate warming, permafrost on the Qinghai–Tibet Plateau is warming and degradating characterized by rising mean annual ground temperature (MAGT) and increasing active layer thickness (ALT). The MAGT at 6 m depth have increased by about 0.43 ^∘^C from Touerjiu Mountains to Fenghuo Mountain during the past decade^3^. The permafrost along the Qinghai–Tibet Highway (QTH) is significantly degraded than in other regions, resulting in an increasing ALT. The averaged increase rate of ALT is 7.5 cm/yr, with a range of 2.1–16.6 cm/yr during the past decade on the QTH^3,4^. Thermokarst is one of the most common manifestations of permafrost degradation, and the ice-rich permafrost thawing leads to surface collapse. This can substantially affect global hydrology^5^, global carbon emissions^6,7^, and infrastructure stability^8^.

Thermokarst lake is one of the most obvious characteristics of the thermokarst landform, which is widely distributed in the permafrost region of the Qinghai–Tibet Plateau. Thermokarst lake, also called thaw lake, tundra lake, thaw depression, or tundra pond^9^, refers to a body of shallow freshwater. Thermokarst lakes in permafrost regions across the Arctic have shrunkwith significant decrease in number and area over the past decade^10,11^. In western Alaska, the expansion of the thermokarst lake occurred parallel to the drainage. Drainage events always occurred in lakes larger than 1 ha (1 ha = 10,000 m^2^), and the area decreased by approximately 151.8 km^2^ between the 1970s and 2010s. However, small and medium lakes have increased in size by 119.9 km^2^ due to drainage from large lakes^12^. The thermokarst lakes on the Qinghai–Tibet Plateau have presented an expansionary trend, and the number of thermokarst lakes in the Beilu River Basin increased by 534, and the size increased by 4.1 km^2^ between 1969 and 2010^13^. About 250 thermokarst lakes were distributed along the Qinghai–Tibet Railway between Kunlun Mountain and the Fenghuo Mountain, with a total area of about 139 $\times$ 10^4^ m^2^^14^.

Thermokarst lake plays an essential role in the ecological environment and infrastructure stability. The permafrost region covers a quarter of the Northern Hemisphere land area with the largest land organic carbon^15^. Abrupt permafrost thawing accelerates organic carbon pool decomposition and releases greenhouse gases (CO_2_ and CH_4_)^16,17^. By analysis of the modeling studies, the amount of carbon released into the atmosphere by the thermokarst lake could double by the end of the century^18^. In addition, organic matter dissolved in thermokarst lake can have significant effects on the diversity and metabolism of bacterial communities, which further increase carbon emissions from permafrost^19^. The thermokarst lake also has an important influence on the stability of the QTH. Lateral thermal erosion causes the permafrost temperature to rise, resulting in subgrade subsidence, deformation, and cracks^20^.

Thermokarst lakes were mainly small sizes with high dense distribution on the Qinghai–Tibet Plateau, so high-resolution images are required to accurately extract thermokarst lake accurately. Thereby, many previous studies on thermokarst lake were mainly limited to small region due to hard obtention of high-resolution image^13^, which was not beneficial to the study of the large-scale spatial distribution and influencing factors of thermokarst lake. So, the freely available Sentinel-2 (S2) images were essential to analyze thermokarst lakes along the QTH, which provided better understand of permafrost degradation in response to future climate and environmental changes. The purpose of this study are to analyze development characteristics, long-term change trend and further to explore the influencing factors along the QTH using S2 and Landsat images. The objectives of this study were to (1) determine the size and spatial distribution of thermokarst lakes on the QTH with 10 m spatial resolution, (2) compare of long-term changes and regional differences in thermokarst lakes along the QTH, (3) explore the factors that influenced spatio-temporal characteristics of thermokarst lake based on permafrost, geographic, climatic and vegetation etc.

## Study area and method

### Study area

**Figure 1:**
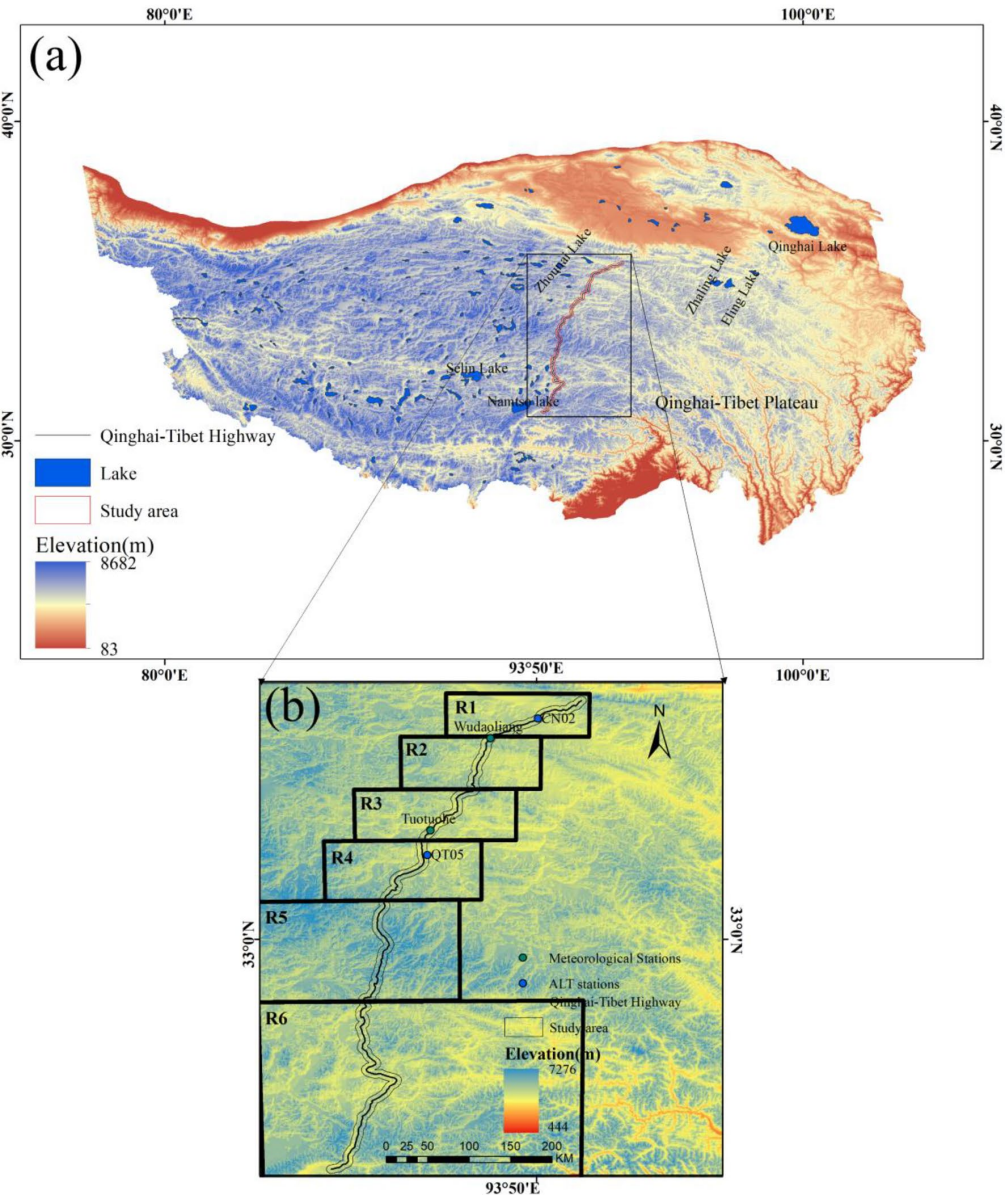
(**a**) Qinghai–Tibetan Plateau (**b**) Study region and sub-region. The map was created using ArcMap 10.8.

The study area is the Golmud-Lhasa section of QTH (Fig. 1). The study region is at an elevation of 4334 m–5621 m, with the average value of 4741.24 m. The QTH passes through a plateau continental climate with the air temperature ranging from − 14.2 ^∘^C in winter to 8 ^∘^C in summer. The average annual precipitation was 303.15–307.42 mm during 1970–2020, with the most precipitation of 503 mm in 2009 and the least of 136.3 in 1984. The average annual evaporation was 1292.92–1587.93 mm between 1988 and 2016, with the most evaporation of 1923.4 mm in 1994 and the least of 1061.9 mm in 1989. The vegetation types along the QTH include alpine meadow, alpine steppe and sparse vegetable. Permafrost was well developed, stretching 531 km from Xidatan to Liangdaohe, with a coverage of 427,915.18 ha (54.43%) of the QTH. The permafrost in the Hongliang River, Beilu River, and Fenghuang Mountain areas was high ice content. The permafrost in the Hongliang River region was mostly ice-rich and soil ice layer, with an upper permafrost limit of about 2.8–3.5 m and MAGT of about − 0.3 to − 2.5 ^∘^C. The permafrost in the Beilu River region was a maximum thickness of 80 m and MAGT of − 0.5 to − 2.0 ^∘^C. The permafrost thickness in Fenghuang Mountain of about 60–120 m and MAGT of − 1.55 ^∘^C. The effect of global warming and the temperature of the asphalt pavement caused the permafrost layer under the roadbed to frequently and rapidly move down^21,22^. The MAGT have increased by 0.12–0.67 ^∘^C at 6 m depth, and exceeded − 1 ^∘^C at 15 m for half of the permafrost^3,23^. The average ALT was about 2.41 m with a range of 1.32–4.57 m between 1995 and 2007^24^. Based on differences in climate, topography, geology, hydrology, lake distribution, and permafrost conditions. The study area was divided into six sub-region R1, R2, R3, R4, R5, R6.

### Data sources

#### Satellite images for thermokarst lake extraction

The S2 and Landsat images are used to delineate thermokarst lakes, which can be freely acquired from the ESA Copernicus Open Access Hub (https://scihub.copernicus.eu/) and the U.S. Geological Survey (https://earthexplorer.usgs.gov/), respectively (Table S1). The S2 satellites are comprised of S2A and S2B, equipped with a MultiSpectral Instrument that acquires 13 spectral bands ranging from visible and near-infrared to shortwave infrared wavelengths. The satellites provide images with spatial resolutions of 10 m (three visible bands and a near-infrared band), 20 m (six red-edge/shortwave infrared bands), and 60 m (three atmospheric correction bands), with a ScanSAR wide of 290 km. S2A and S2B satellites have a revisit period of 10 days near the equator and 6 days at mid-latitudes. Landsat-5 has Multispectral Scanner (MSS) and Thematic Mapper (TM) sensors, which ceased operation in 1995 and 2011, respectively. The Landsat-5 TM has a 16-day revisit cycle and a ScanSAR wide of 185 km. Landsat-5 has seven bands, which has a spatial resolution of 30 m, except for B6 with 120 m. Landsat-8 has Operational Land Image (OLI)(B1–B9 bands) and Thermal Infrared Sensor (TIS)(B10 B11 bands) sensor. The B8 is the panchromatic band (15 m), the spatial resolution of the other bands is 30 m, and B10 B11 with a spatial resolution of 100 m. The Landsat-8 OLI sensor has a 16-day repetition cycle and a ScanSAR wide of 185 km. The S2 MSL2A and Landsat Collection 2 Level-1 dataset have finished the geometrical correction and radiative correction of the image. Additionally, The Normalized Difference Water Index.(NDWI) was used to identify lake boundaries, which was calculated in ArcGIS software using the near infrared (NIR) and green bands.

1 $$\begin{aligned} NDWI=\frac{GREEN-NIR}{GREEN+NIR} \end{aligned}$$

Bands 3,8 of S2 and 2,4 of Landsat are green and NIR bands, respectively. The S2 and Landsat images were selected in October with less clouds. If there was insufficient data in October, the data was extended to August and September. Water bodies with an area large than 300 ha were excluded to reduce the possibility of other lakes being classified as thermokarst lakes. Considering that at least 5–6 pixels with 10 m spatial resolution were required to reliably identify the lake according to the mixed pixel decomposition theory^25^, so the minimum area of thermokarst lake is limited to 0.05 ha. To ensure the accuracy of the boundary extraction of thermokarst lakes, we compared the number and area of lakes obtained from visual interpretation of satellite images with the results of NDWI extraction. Some of the thermokarst lakes in the six subregions were manually extracted, and the number of manually extracted thermokarst lakes in the six sub-regions was 1068, with a total area of 1135.63 ha. Compared with the 1197 thermokarst lakes (1265.73 ha) extracted by NDWI, the error was less than 12%. To demonstrate the accuracy of NDWI in large lakes. 15 thermokarst lakes with areas of 10–300 ha were selected to proceed manual visual interpretation and compared them, and the total area extracted by NDWI (513.52 ha) was 10% different compared to the manual visual interpretation results (463.35 ha). Therefore, NDWI can be used accurately for water body extraction.

#### Permafrost and climate data

The spatial distribution of permafrost type, MAGT and ground ice content over the study region and long-term, averaged ALT along the QTH were provided by the Cryosphere Research Station on the Qinghai–Tibet Plateau, Chinese Academy of Sciences. The two observation stations in the study region were located in Suonandajie (CN02) (93.60^∘^ E, 35.43^∘^ N, 4488 m) and Kaixinling (QT05) (92.40^∘^ E 33.95^∘^ N 4652 m). Soil temperature was observed at different depths, which was monitored by 105T thermistor sensors with observation accuracy of 0.1 ^∘^C. The observation instruments were connected to CR1000 logger (Campbell Company). The time was based on Beijing, and data was obtained once every 0.5 h (QT05) and 2 h (CN02). ALT was determined by measuring the maximum depth of the 0 ^∘^C isotherm observed from the soil temperature profile. The air temperature, precipitation, and evaporation recorded from Tuotuohe (TTH)(92.43^∘^ E, 34.22^∘^ N, 4533.1 m a.s.l.) and Wudaoliang (WDL) (93.08^∘^ E, 35.22^∘^ N, 4612.2 m a.s.l.) were obtained form the National Meteorological Information Center (NMIC), China Meteorological Administration (CMA) (http://cdc.cma.gov.cn).

### Study methods

#### Univariate linear regression analysis

Univariate linear regression analysis forecasting method, was based on the correlation between the independent variable x and the dependent variable Y, the establishment of a linear regression equation of x and Y for forecasting.

2 $$\begin{aligned} y=A+Bx \end{aligned}$$

Univariate linear regression equations can be derived using n sets of independently collected data.

3 $$\begin{aligned}{} & {} B=\frac{\sum (x_i-\overline{x}) (y_i- \overline{y})}{\sum (x_i- \overline{x} )^2} \qquad \end{aligned}$$

4 $$\begin{aligned}{} & {} A=(y - B \overline{x}) \end{aligned}$$

In order to be the optimal straight line, it is necessary to determine A and B. According to the principle of differential theory, A and B were calculated according to Eqs. (3) and (4), respectively.

#### Mann–Kendall trend test

The Mann–Kendall trend test (M–K) is a widely used statistical test for predicting the long-term trends of meteorological elements such as temperature, precipitation, and barometric pressure. The non-parametric test, also known as the distribution-free test, does not necessarily have the characteristics of normal distribution of the change elements, and will not be affected by a small number of outliers, and has a high degree of quantification, a wide range of detection, low interference, simple calculation. Therefore, it is suitable for the analysis of the trend of the change of the characteristics of the non-normal distribution of the change elements such as meteorological elements. A1, A2,..., An are the time series variables, n is the number of samples, then the statistic S can be defined as:

5 $$\begin{aligned} S=\sum _{i=2}^{n} \sum _{j=1}^{i-1}sgn(A_i-A_j) \end{aligned}$$

(5) Eq. sgn represents the sign function; S represents the normal distribution, the mean is equal to 0, and its variance is calculated as shown in Eq. (6)

6 $$\begin{aligned} \displaystyle sgn(A_i-A_j )= \left\{ \begin{array}{l} 1,\ A_i-A_j > 0 \\ 0,\ A_i-A_j=0 \\ -1,\ A_i-A_j<0 \end{array}\right. \end{aligned}$$

The corresponding Z values for different S intervals in the Mann-Kendall statistic formula are:

7 $$\begin{aligned} \displaystyle Z= \left\{ \begin{array}{l} (S-1)\sqrt{(Var(s)} ),\ S<0 \\ \ 0,\ S=0 \\ (S+1)\sqrt{(Var(s)}),\ S>0 \end{array}\right. \end{aligned}$$

Mutation analysis was performed using the M–K test. In the M–K trend test, the hypothesis is not valid if |Z| $\ge$ Z1-a/2 for a specific confidence level a. When Z > 0, it means an increase in magnitude, and when Z $<0$, it means a decrease in magnitude. When Z>0, it means that the amplitude is increasing, and when Z $<0$, it means that the amplitude is decreasing. When |Z| > 1.28, it means the significance test is more than 90%, when |Z| $\ge$1.64, it means the significance test is more than 95%, when |Z| $\ge$2.32, it means the significance test is more than 99%.

#### Number and area density

In order to better analyze the lakes in each subregion, we used number density versus area density for our analysis.

$$\begin{aligned} Number density=\frac{L_1}{L_n} \\ Area density=\frac{L_2}{L_n} \end{aligned}$$

$$L_1=Number\ of\ lakes$$

$$L_n=The \ overall\ size \ of\ the\ study \ area$$

$$L_2=Lake\ area$$

## Results

### Size characteristics and spatial distribution

**Figure 2:**
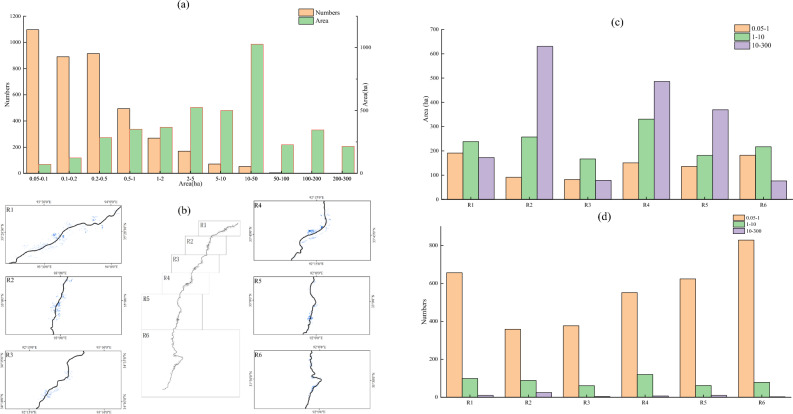
(**a**) The number and area of small, medium, and large thermokarst lakes in the study region in 2021, (**b**) Distribution of thermokarst lakes in sub-regions of the study region, (**c**) The area of thermokarst lakes in sub-regions, (**d**) The number of thermokarst lakes in sub-regions.

Figure 2a shows that 3965 thermokarst lakes were observed in the study region in 2021, with a total area of 4038.6 ha. The thermokarst lakes were divided into small (0.05–1 ha), medium (1–10 ha), and large lakes (10–300 ha). Among these lakes, there were 3396 small lakes (85.65%) with an area of 833.42 ha (23.95%), 510 medium lakes (12.86%) with an area of 1391.13 ha (39.98%), and 59 large lakes (1.48%) with an area of 1814.05 ha (52.14%), respectively (Fig. 2a). The number of small lake (85.65%) was significantly more than medium-large lakes (12.86% and 1.48%), whereas the area of large lake (52.14%) made up a large proportion of 52.14% of the whole surface area.

The size characteristics of the thermokarst lake showed a significant spatial difference in sub-regions (Fig. 2b). The distribution of lake type in all sub-regions were similar to that of overall distributions, with significantly higher numbers of small lakes than medium-large lakes in each sub-region. However, the surface distribution of some sub-regions did not exactly follow the general trend, with large lakes dominating. In the R1, the three types of lakes were roughly evenly distributed; the area of medium size lakes (39.61%) was slightly larger area than that of small lakes (31.73%) and large lakes (28.65%) (Fig. 2c). In the R3, thermokarst lake was mainly dominated by medium lakes, accounting for 50.86% of the total size. Lake size in R6 was predominantly small-medium lakes, with proportions area of 182.09 ha (38.30%) and 217.04 ha (45.65%), respectively (Fig. 2c). The proportion of lake area in R2, R4 and R5 were all significantly higher for large lakes than for small-medium lakes in total. The number density of R1, R2, and R4 was greater than that of the total density. The number density of R1 was the largest among the sub-regions at approximately 0.0071 (Table 1). However, the area density of R1 was only 0.0055 as the fourth indicating that R1 were mainly small-medium lakes. The surface density of the lakes in the R2 area was 0.013, which was significantly higher than the rest of the sub-regions, indicating that the lakes in R2 were more developing than the rest (Table 1).

**Table 1:** Number and area density of whole and sub-regions.

	Number of lakes	Number density	Lake area (ha)	Area density
R1	765	0.007054	602.13	0.005552
R2	473	0.006496	979.04	0.013446
R3	442	0.004829	327.93	0.003583
R4	679	0.006920	967.43	0.009860
R5	696	0.004922	686.64	0.004856
R6	910	0.003220	475.43	0.001682
R7	3965	0.004988	4038.6	0.005081

The analysis showed that the number of small lakes in each sub-region exceeded 75%, which was far more than that of medium and large lakes (Fig. 2d). The number of small lakes is the largest of the total R6 lakes accounting for 90.99%, however, whose area only occupied for 38.30%. The development of lake in, R2 and R4, whose total lake area accounted for 48.20% of the overall lake surface area (Fig. 2c), was better than the others. The surface area of large lakes dominated in R2, R4, and R5, accounted for 64.44%,50.23%, and 53.83%, respectively (Fig. 2c). The lakes in R1, R2, and R4 highly developed, with significantly higher numbers and area densities than those of the overall density (Table 1).

### Change trend

**Figure 3:**
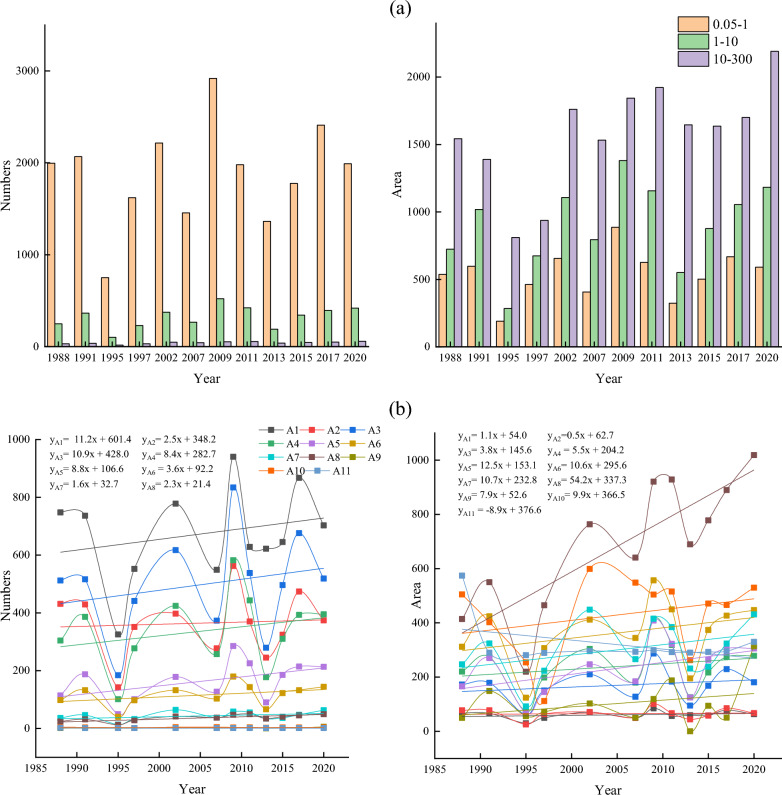

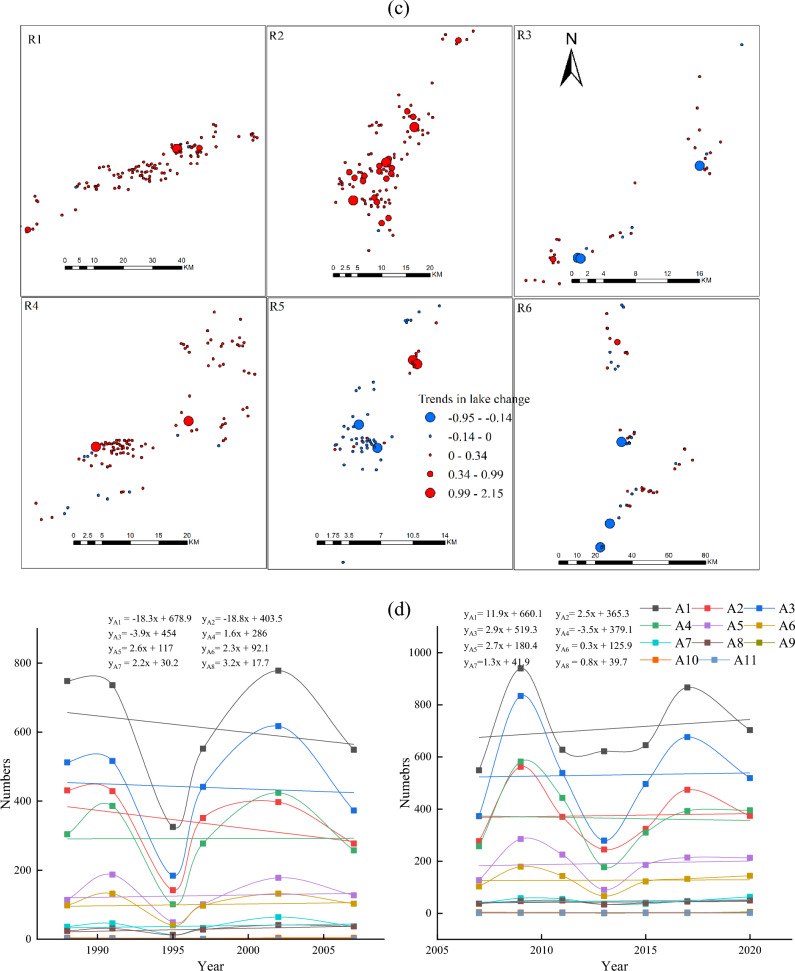

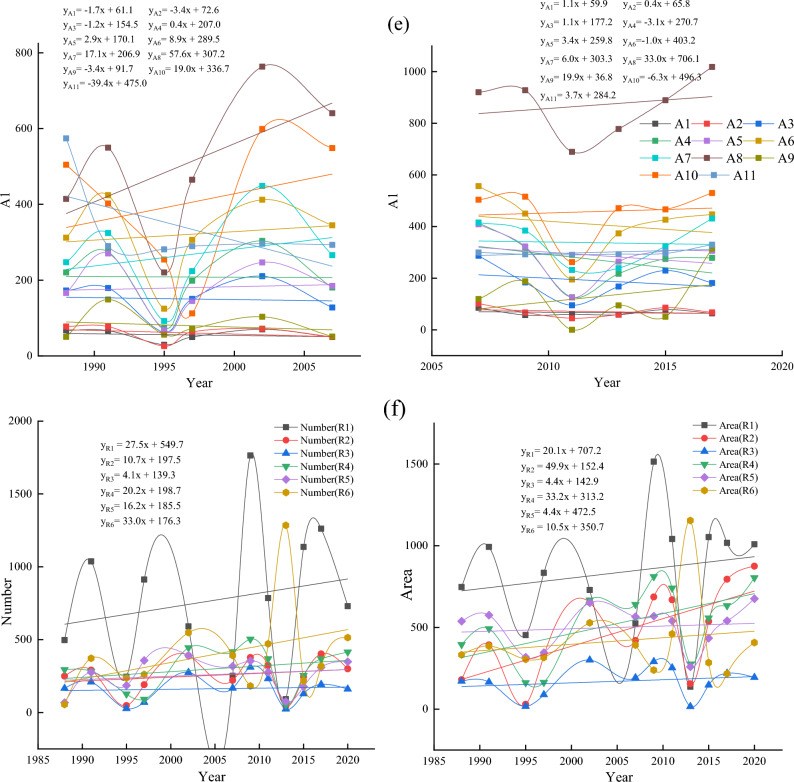

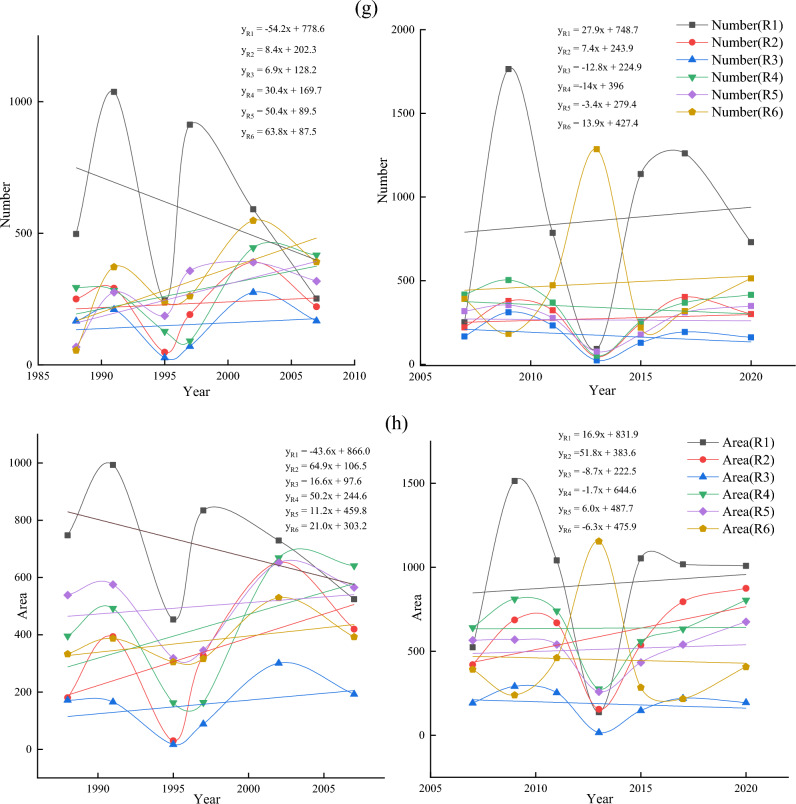
(**a**) Number and area of thermokarst lakes in different years from 1988 to 2020, (**b**) Trends in medium and large lakes in the subregion 1988–2020, (**c**) Trends in the number and area of different types of thermokarst lakes from 1988 to 2020, (**d**) Trends in the number of different types of thermokarst lakes from 1988 to 2007 and 2007 to 2020, (**e**) Trends in the area of different types of thermokarst lakes from 1988 to 2007 and 2007 to 2020, (**f**) The change rate of lake number and area in different sub-regions during 1988–2020, (**g**) Change rate of lake number in different sub-regions during 1988–2020, (**h**) The change rate of lake area in different sub-regions during 1988–2007 and 2007–2020.

Figure 3a shows that from 1988 to 2020, there was an overall increase in both lake number and area, with an increase of 195 (8.58%) in the number and a significant increase of 1160.19 ha (41.36%) in the area. The area of small lakes only increased by 52.92 ha (9.85%). Whereas the total area of medium-large lakes increased by 458.64 ha (63.30%) and 648.63 ha (42.04%), respectively (Fig. 3a). Thermokardst lakes were divided into eleven classes in order to analyze variation difference for different lake sizes in detail: A1 (Area = 0.09 ha), A2 (0.09 ha < Area $<=0.2$ ha), A3 (0.2 ha < Area $<=$ 0.5 ha), A4 (0.5 ha < Area $<=1$ ha), A5 (1 ha < Area $<=$ 2 ha), A6 (2 ha < Area $<=$ 5 ha), A7 (5 ha < Area <=10 ha), A8 (10 ha < Area <= 50 ha), A9 (50 ha < Area <= 100 ha), A10 (100 ha <Area<= 200 ha), A11 (200 ha < Area <= 300 ha) (Fig. 3b). Between 1988 and 2020, except the size of A11 the areas of other lake sizes were increasing. The A8 expanded significantly more than other growth types, with 54.2 ha/yr. There was a significant change in the growth rate of small-medium lakes, with the number of lakes in A1 and A3 increasing by more than 10 each year (Fig. 3b). The rate of change of the number of lakes (< 50 ha) was much more significant those over 50 ha. The small-medium lakes were far less stable than large lakes, so there was a substantial change in the number of small-medium lakes. However, the rate of change in the area of large lakes was significantly higher than that of small-medium lakes (Fig. 3b).

The emergence and expansion of thermokarst lakes significantly influence the increase in the count of and area. Statistical analysis of lake emergence and expansion rates in two phases (1988–2007 and 2007–2020). The results showed that other classes’ lake emergence and expansion rates differed significantly between the two phases. Small lakes’ emergence and expansion rates in most of the small lakes in Phase II were substantially higher than those in Phase I. The two phases were similar in medium-sized lakes, and Phase II was smaller than Phase I in large-sized lakes. Some small-sized lakes in Phase I showed a noticeable decreasing trend, while the lakes in Phase II kept increasing (Fig. 3d). Small lakes (A1, A2, and A4) had much higher rates of change in numbers than did the other lake types, with rates of change ranging from − 18.3 each year, − 18.7 each year, and 1.6 each year to 11.9 each year, 2.5 each year, and − 3.5 each year for A1, A2, and A4, respectively (Fig. 3d). The expansion rate of large lakes (A8) was much higher than that of other lake classes, and Phase II expanded at a slower pace than Phase I, increasing from 57.6 to 33.0 ha/yr (Fig. 3e).

The analysis of the trends of medium-large lakes in each sub-region from 1988 to 2020 indicated that the overall trends of medium-large lakes in sub-regions R1, R2, and R4 had increased (Fig. 3c). Most of the lakes in R1 showed a growing trend, with the largest annual growth rate in 1.14 ha/yr. The growth trend of lakes in R2 was much larger than that in other regions. There were 17 lakes with growth rates of 0.34–0.99 ha/yr and three lakes larger than 0.99 ha/yr, with the most significant change in trend showing an annual growth of 2.04 ha/yr (Fig. 3c). Most of the lakes in R3 were increasing, but the trend of area increase was slight, and generally less than 0.34 ha/yr. Although there was an increasing trend in the number of lakes, the decreasing area was significant than 0.21 ha/yr (Fig. 3c). A few lakes in the southern part of R4 showed a decreasing trend, with the two most notable annual growth rates of 2.13 ha/yr and 2.15 ha/yr, respectively (Fig. 3c). The overall noticeable decline in the R5, with the most significant decreasing trends of 0.17 ha/year and 0.14 ha/yr. However, there were two lakes with significant upward changes of 1.30 ha/yr and 1.79 ha/yr, respectively (Fig. 3c). R6 had a larger area and the medium-large lakes in the north had close to the same trend in area. In the central and southern, 19 lakes showed an increasing trend, and 14 lakes showed a decreasing trend. However, the decrease was much greater than the increase, with the most significant lake trends being − 0.23 ha/yr and − 0.15 ha/yr, respectively (Fig. 3c). During 1988–2020, the area of thermokarst lakes in the permafrost generally showed an increasing trend, and only a few lakes decreased.

Thermokarst lakes showed an increased trend from 1988 to 2020 (Fig. 3b). However, thermokarst lakes’ emergence and expansion rates changed significantly at different stages in different sub-regions (Fig. 3f). The number of lakes in R1 increased in both phases. However, the Phase I lake area growth rate decreased, while Phase II increased linearly (Fig. 3g,h). In both phases, the rate of change in the number and area of lakes in R2 has remained essentially constant, with a linear upward trend (Fig. 3g,h). The number and area of lakes in R3 showed a decreasing trend, changing from 6.8 each year and 16.6 ha/yr to − 12.8 each year and − 8.7 ha/yr, respectively. The number and area of thermokarst lakes in R4 increased significantly in Phase I than in Phase II. Especially the size increase rate changed from positive (50.2 each year) to negative (− 1.7 each year) (Fig. 3g,h). The number and area of lakes in R5 showed a decreasing trend. The rate of increase in the number of lakes in Phase I was twice as much as in Phase II, with a significant change in surface area from 50.97 to − 3.4 ha/yr. (Fig. 3g,h). The number of R6 lakes showed an increasing trend, but the growth rate of lakes in Phase I was significantly higher than in Phase II, which changed from 63.8 each year to 13.9. The area was in a decreasing trend, with a positive growth rate (21.207 ha/year) to a negative growth rate (− 6.3 ha/year) (Fig. 3g,h). In the overall analysis, the number and area of thermokarst lakes increased in each sub-region. However, the analysis of the different phases showed that not all regional ones increased at different times and locations. The most noticeable changes were in R1 and R4, where R1 changed from an overall decrease in number and area of lakes in Phase I to an overall increase in which in Phase II. In contrast, the lake area in R4 changed from a general rise to an overall decrease.

Thermokarst lakes in different sub-regions were analyzed using Landsat data from 1988 to 2020. Statistical analysis showed that the highest percentage of the number and area of thermokarst lakes in the R1 were 730 and 1008.63 ha, accounting for 29.57% and 25.43% of the total thermokarst lakes, respectively. The R3 had the lowest ratio of the entire thermokarst in the number and size of lakes, and the number and size of lakes were 161 (6.52%) and 194.22 ha (4.90%), respectively. Among all sub-regions, the number of thermokarst lakes increased fastest in R1, and the surface increased fastest in R6. The number and area of thermokarst lakes are increasing in different classes and sub-regions. The increase in the number and size of different types of thermokarst lakes in Phase I was much smaller than in Phase II. In different sub-regions, the number of thermokarst lakes in Phase I varied much more than that in Phase II besides the R1, but the change rate of lake size in Phase II mainly increased.

### Seasonal variation

**Figure 4:**
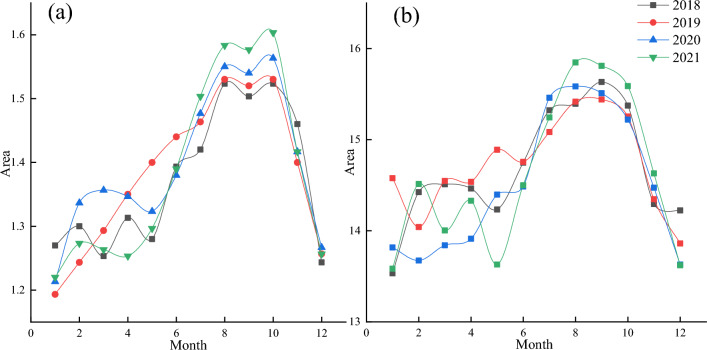
(**a**) Lake A four years of seasonal variation, (**b**) Lake B four years of seasonal variation.

Two thermokarst lakes in the Beiliu River were selected for seasonal variation analysis. The thawing time of the two thermokarst lakes changed by latitude, climate. Analysis of the seasonal variation of the two lakes indicated that lakes A and B were in development between 2018 and 2021. The surface area of both lakes had a notable increase each year. Melting ground ice was attributed to increasing temperatures and provided water for lakes A and B, increasing surface area. Lake A reached its first peak in August, while the surface area of the lake decreased as water seeped into the ground. However, the area of Lake A peaked at the beginning of October due to the accelerated melting of the ground ice caused by the continued increase in temperature (Fig. 4a). Lake B started to melt in mid-June and the area reached its maximum in early September, after which the area declined slightly and remained stable (Fig. 4b). For 2018–2020, Lake A and B have an area growth rate of 0.042 ha/yr, 0.14 ha/yr (June–November) in the summer and autumn, respectively. Precipitation is a crucial resource affecting the water balance of the thermokarst lake. Gao et al analyzed the precipitation near the thermokarst lake separately. It was found that the anions and cations follow the same trend as the electrolytes in the lake, indicating that precipitation was an essential source of water balance in the thermokarst lake^26^. The permafrost at both lake locations reaches the zero curtain of the fall freezing process after the beginning of October, and the thermokarst lake was equivalent to a heat source exothermic to the surrounding frozen active layer. The combined effect of the external environment and the active layer caused a sharp decrease in the lake area after October. By analyzing seasonal variations of the two lakes, it was found that precipitation and melting of ground ice provide the conditions for the water balance and area increase of the thermokarst lake. However, permafrost characteristic of bi-directional freezing causes the lakes to freeze rapidly.

### Influencing factors

#### Permafrost characteristic

**Figure 5:**
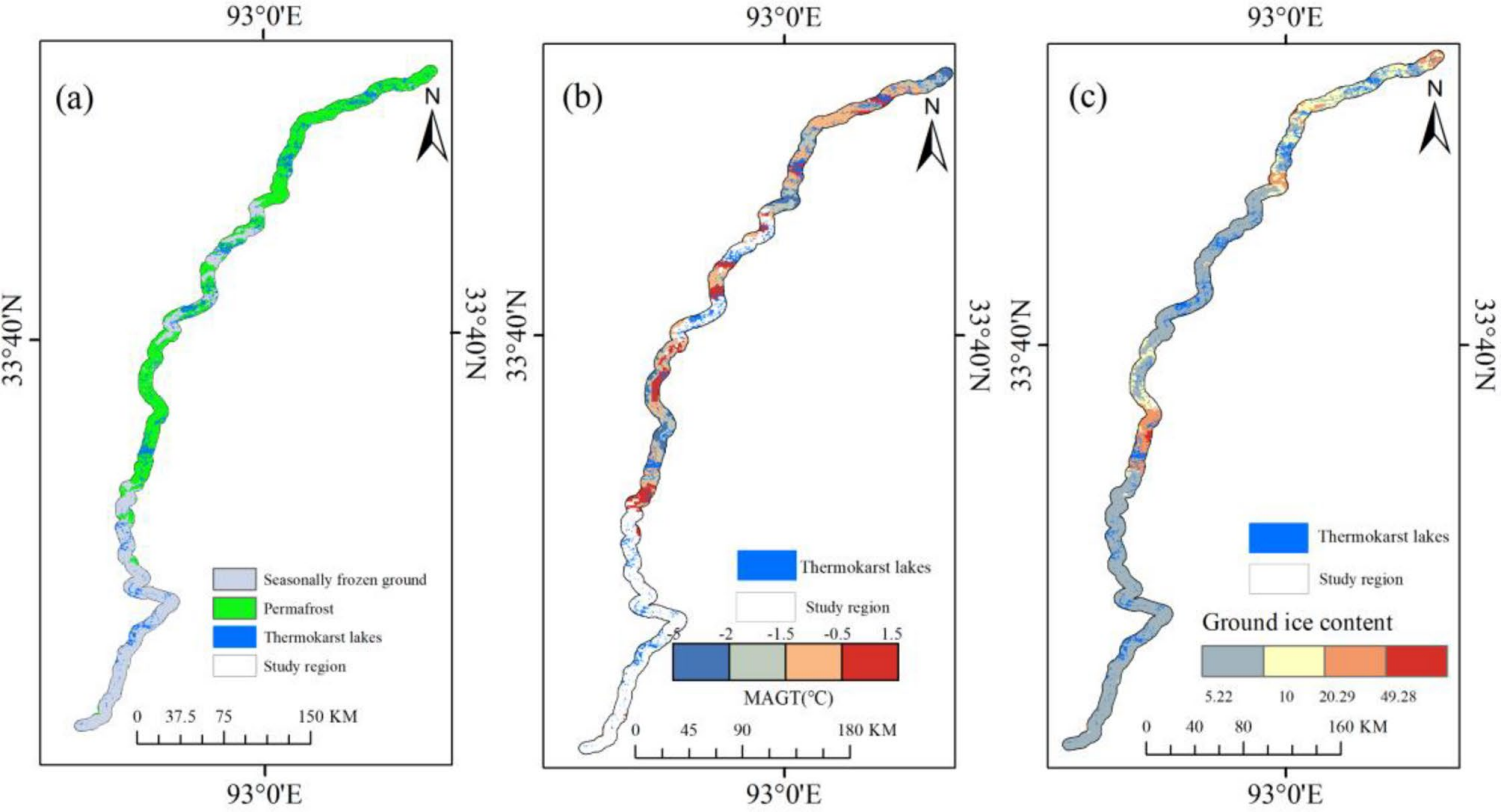
(**a**) Permafrost and seasonally frozen permafrost, (**b**) MAGT, and (**c**) Ground ice content.

Permafrost type, MAGT, and ground ice content were analyzed to determine the relationships between permafrost characteristic and thermokarst lake distribution (Fig. 5a–c). The study region was across the permafrost with a coverage of 427,915.18 ha (54.43%) (Fig. 5a). The count and surface of thermokarst lakes in permafrost were 2054 and 2321.09 ha, which accounted for 51.80% and 57.47% of the total number and size of lakes, respectively (Fig. 5a). The number and surface of lake in the warm permafrost region (MAGT > − 1.5 ^∘^C) were 1879 and 2214.69 ha, accounting for 91.94% and 95.42% of the total number and surface of lakes, respectively (Fig. 5b). The number and area of lakes in permafrost region withlow ground ice content (> 10 $\times$ 10^6^ m^3^) were 3521 (88.80%) and 3816.66 ha (94.50%), respectively (Fig. 5c). Number densities of thermokarst lakes in sub-region was with value of 0.007 for R1 and was lowest for R6 with value of 0.003 (Table 1). These results indicated that thermokarst lakes were widely developed in warm permafrost region with low ground ice content. This was because the magnitude of ALT of warm permafrost was significantly greater than that of the cold permafrost^4^, which has accelerated the melting of ground icein warm permafrost, and provided more water for the formation of thermokarst lakes.

#### Terrain and vegetation

**Figure 6:**
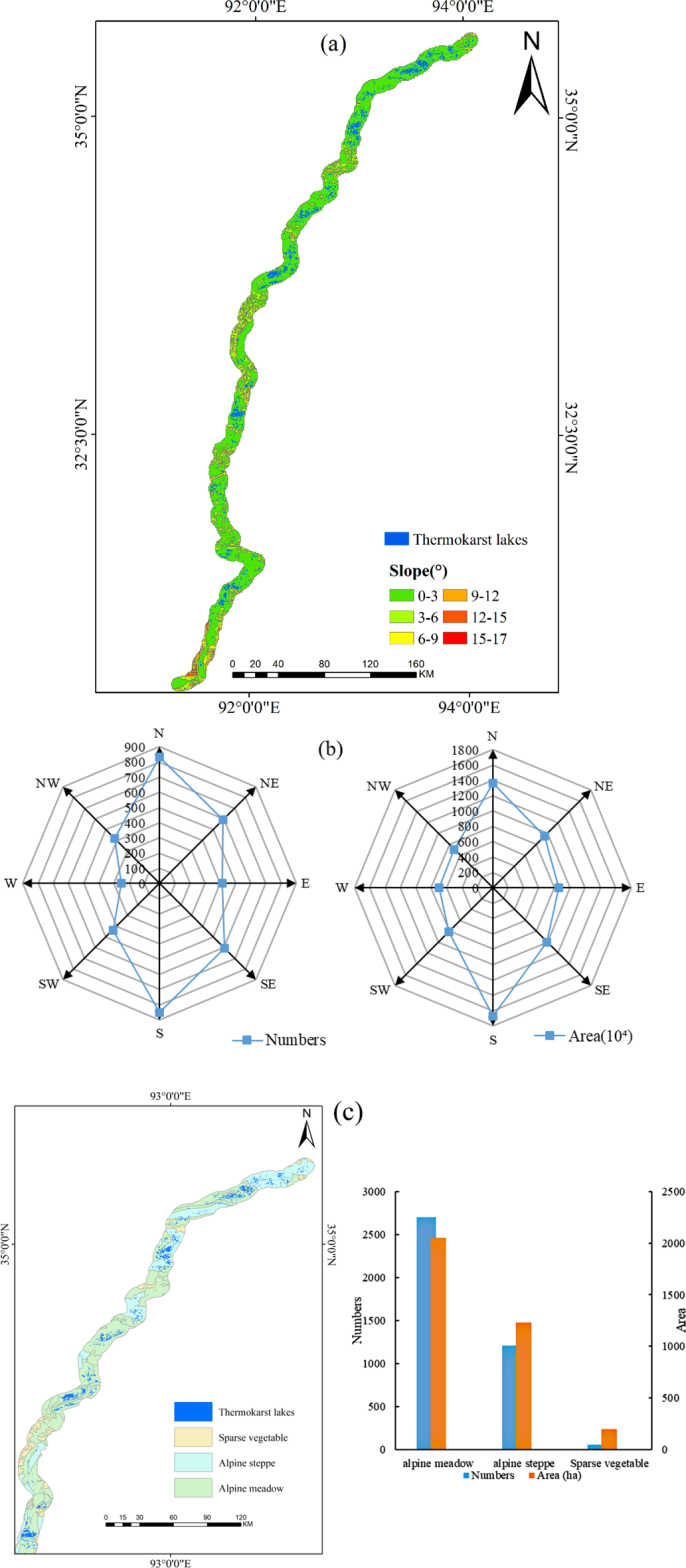
(**a**) Distribution of thermokarst lakes at different slopes, (**b**) number and area of thermokarst lakes at different slope aspects, (**c**) vegetation types and lake distribution in Qinghai Province.

More than 90% of the lakes were mainly distributed in elevation range of 4300–5000 m, in which number and area of the lakes were 3738 and 3922.73 ha, accounting for 94.27% and 97.13% of thermokarst lakes, respectively. The slope in study region was divided into 0–3^∘^, 3–6^∘^, 6–9^∘^, 9–12^∘^, 12–15^∘^ and 15–17^∘^. Thermokarst lakes had a significant develop-ment on slopes less than 3^∘^, with 3932 (99.17%) and 3976.72 ha (98.47%) of the number and area of total lakes, respectively (Fig. 6a). The study area was a north-south shape with very flat terrain. Thermokarst lakes mainly occurred in the south and southeast directions, with 33.06% and 33.05% of the number and size, respectively (Fig. 6b). The vegetation types contained alpine meadow (63.40%), alpine steppe (30.56%) and sparse vegetation (Fig. 6c). The proportion of thermokarst lake in alpine meadow was the largest, and the number and size of lake were 2704 (68.20%) and 2422.99 ha (60.00%), respectively (Fig. 6c). Followed by alpine steppe, the number and size of thermokarst lake was 1206 (30.41%) and 1413.78 ha (35.01%), respectively (Fig. 6c). The results showed that the terrain was an important factor that contributed to the development of thermokarst lake; thermokarst lakes were widely develop in the elevation range of 4300–5000 m, on slope less than 3^∘^, and south and south-east directions. This was attributed that the flat terrain stored water and the sun radiation accelerated ground ice melting of ground ice, leading to the development of thermokarst lakes. This was well verified by that thermokarst lakes were significantly developed in the alpine meadow region.

#### Climate and permafrost conditions

**Figure 7:**
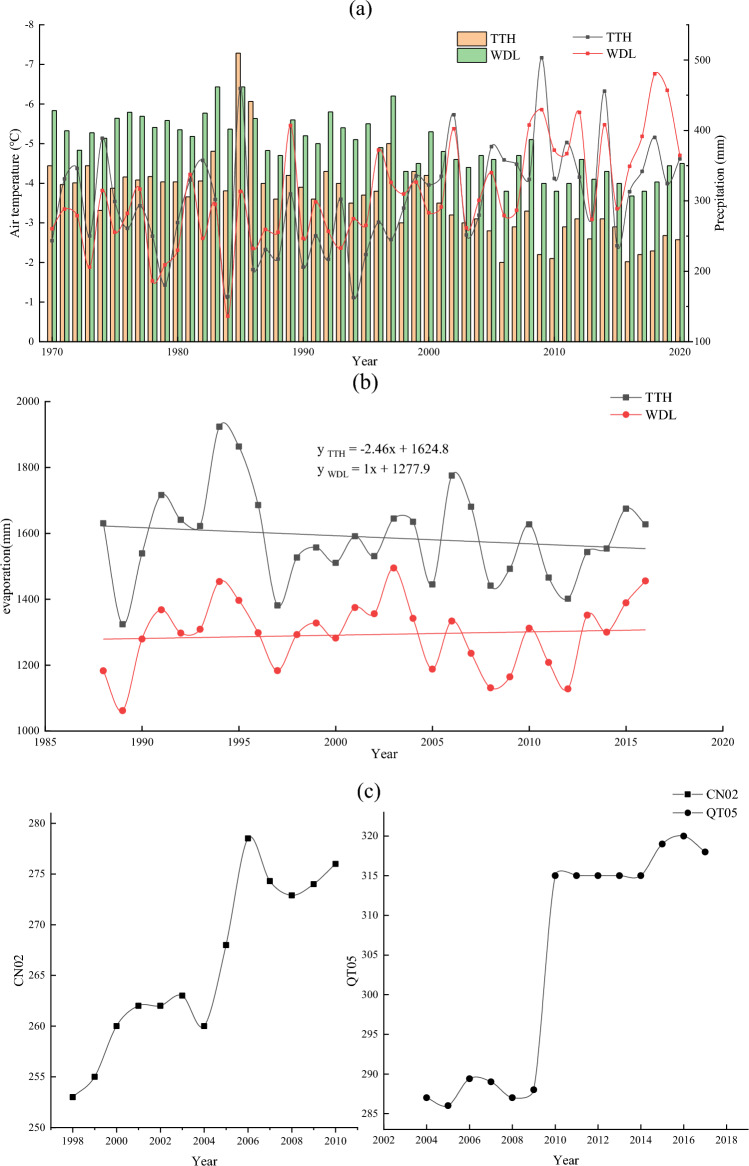
(**a**) Mean annual air temperature and annual precipitation over the past 50 years, (**b**) annual evapora-tion over the past 30 years, (**c**) ALT change rates for CN02 and QT05.

The analysis of the climate data from TTH and WDL meteorological stations indicated that the mean annual air temperature and annual precipitation were increasing from 1970 to 2020. The air temperature has risen by 2 ^∘^C and 1.3 ^∘^C, with growth rates of 0.04 ^∘^C and 0.03 ^∘^C, respectively (Fig. 7a). The annual precipitation has increased by 116.4 mm and 104.2 mm, and rates of 1.76 mm/yr and 3.07 mm/yr, respectively (Fig. 7a). Precipitation was decreasing and then increasing, which was consistent with thermokarst lakes were shrinking and then expanding. Particularly, precipitation in 1995 was 136.3 mm and 223.6 mm, the almost lowest number since the past 50 year. This was corresponding to the lowest values both area and number of thermokarst lake (Fig. 3b). The significance of temperature and precipitation in both TTH (|Z| = 5.73) and WDL (|Z| = 5.70) was greater than 99%, and the significance of precipitation in both TTH and WDL was also greater than 99%, which proved that there was a significant trend of increasing temperature and precipitation with time. Figure 7b showed that evaporation displayed a notable decreasing trend (− 2.46 mm/yr) at the TTH site and a relatively slow increasing (1 mm/yr) at WDL site during 1988–2016. The increasing evaporation for WDL station was not in accordance with the continuous increase in count and coverage of thermokarst lake in this region. These results indicated that precipitation has a great influence on area and number of thermokarst lake, but the evaporation was not an important factor to enhance lake size. Soil heat fluxes increase due to rising mean annual air temperatures. Changes in soil heat flux were an important cause of changes in soil temperature. Soil temperature increases with increasing soil heat flux, leading to a rise in ALT. Figure 7c showed that the ALT presented a significant increase over the past several decades, ranging from 253 to 276 cm (1.98 cm/yr) for CN02 during 1998–2010 and 287 cm to 318 cm (3.18 cm/yr) for QT05 during 2004–2017. The increase in air temperature led to an increase in the ALT and accelerated the melting of ground ice, which was the main driving factor for thermokarst lake expansion.

## Discussion

### Thermokarst lake potential change trend

**Figure 8:**
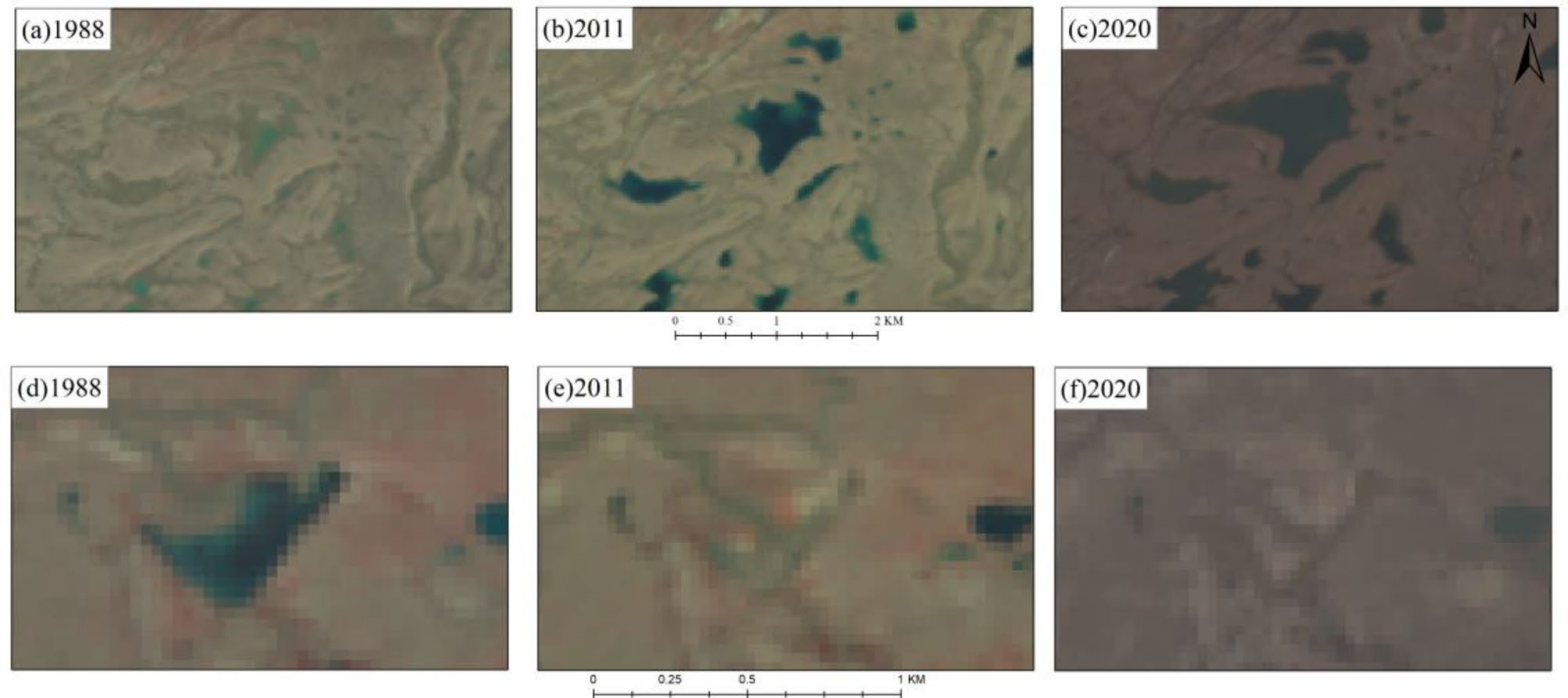
Example of lake changes in the study region from 1988 to 2020. Lake expansion in the study region in (**a**) 1988, (**b**) 2011, and (**c**) 2020. Lake drainage in the study region in (**d**) 1988, (**b**) 2011, and (**c**) 2020.

The thermokarst lakes within a 5000 m range along both sides of QTH were extracted in the study region, indicating that they were developed in ice-rich permafrost region, and experienced the notable expansion between 1988 and 2020 (Fig. 8a–c). This finding was generally consistent with the results of Luo et al.^13^; thermaokrast lakes have increased 534 and 410 ha of the count and size in the Beilu River region between 1960 and 2010, respectively^13^. Although the thermokarst lakes in study region appeared an overall expansion trend, partial lakes have shrunk or even disappeared. The number of thermokarst lake in study region has decreased by 61 (1.54%) with area decrease of $-$ 4.18 ha (0.1%) (Fig. 3c). Luo et al.^27^ also confirmed that the thermokarst lakes were shrinking on the Qinghai–Tibetan Plateau. The lakes area have decreased by 8% of the whole lake, and 6% of lakes have disappeared in the Beilu River Basin^13^. Maybe, as accelerating permafrost degradation,this decrease trend of thermokarst lakes areas in study region will intensify in the future, which was similar to the trend in the circum-Arctic region^5^. The area of thermokarst lake in western Alaska has decreased by 15180 ha in continuous and discontinuous permafrost regions. The reduction in thermokarts lakes may be due to complete thawing of the permafrost, forming open talik that lead to groundwater systems connecting to the permafrost aquifer^28^. The talik connected surface water with groundwater, which has resulted in surface water in-flow to groundwater, accelerating the shrinkage of the thermokarst lake (Fig. 8d–f)^29^. This apparent phenomenon was well verified by Liu et al.^29^; the Yellow River Source Area has reduced 5418 (56.1%) of the whole lake number with 58.63 $\times$ 10^6^ m^2^ (49.0%) of the whole lake area^29^. Therefore, we infer that in the future the thermokarst lake on the Qinghai–Tibet Plateau will be likely to shrink or even disappear in the future, as in the circum-Arctic region.

### Retrogressive thaw slump

Retrogressive thaw slump has occurred in area of high surface water abundance^30^. The thermokarst lake was taken as heat source, which would continue to release heat to the bottom and shoreline of lake, thus accelerating the formation of retrogressive thaw slump. Retrogressive thaw slump was divided into three phases: crack formation, crack expansion and block collapse. The cracks were often developing rapidly during the summer. Thermokarst lake was well-development of retrogressive thaw slump in the same direction as the dominant wind and sunny slope with more radiation^31^. When the ice-rich permafrost melts, the lake shore bounded by the cracks tilts back to the lake and collapses into the water. In Beilu River Basin, 80% of the lake experienced retrogressive thaw slump, with all lake shore setbacks more than 0.5 m, the largest value of which was 1.8 m^32^. In the Central Yakutia, the rate of retrogressive thaw slump was much more than in the Beilu River Basin with a range of 0.5 and 3.16 m/yr^31^. In the Herschel Island coast and the Mackenzie Delta region of Canada, the retrogressive thaw slump rates of the thermokarst lakes were 9.6 m/yr and 1 m/yr, respectively^33,34^. Therefore, retrogressive thaw slump was the main reason for the expansion of thermokarst lakes.

### Frost mound

The melting of the frozen mounds was one of the main reasons for the formation of thermokarst lakes. Frost mounds were formed by groundwater freezing and rising, which divided into opening and closing types. The opening type of frost mounds was formed in discontinuous permafrost areas or areas with thin continuous permafrost, where surrounding groundwater frozen and then raised. The closing frost mounds were developed via supra-permafrost water. There was a significant development of frost mounds on the QTH, with many frost mounds observed in the Wuli region, reaching 9–10/km^2^ in the densest areas^35^. As the south-facing slope of frozen mound was exposed to more radiation, as well as the caves that were dug by alpine animals around frozen mounds^36^, the ice inside the frozen mound begun to melt and the melwater then converged into the low-lying area eventually form thermokarst lakes.

## Conclusion

In this paper, based on S2 (2021) and Landsat (1988–2020) images, thermokarst lakes within a 5000 m range along both sides of QTH were extracted to analyse their spatio-temporal variations. The results showed that the number and area of thermokarst lake in 2021 were 3965 and 4038.6 ha with an average size of 1.0186 ha. Although small thermokarst lakes (3396) accounted for 85.65% of the entire lake count, the surface was only covered by 20.64% of the whole lakes. The 59 large thermokarst lakes covered a surface of 1814.05 ha, accounting for a large proportion of 44.92% of the whole lake area. In all sub-regions, the number of small lake far exceeds 75% of the total lake number in each sub-region. R1 sub-region (around Wudaoliang region) had the maximum number density of thermokarst lakes with 0.0071, and R6 sub-region (around Anduo region) had the minimum number density with 0.0032. Thermokarst lakes were mainly distributed within elevation range of 4300 m–5000 m a.s.l., occupying for 94.27% (3738) and 97.13% (3822.73 ha) of the total number and size, respectively. They were observed on flat terrain with slopes less than 3^∘^, accounting for 99.17% (3932) and 98.47% (3976.72 ha) of the whole count and coverage, and in the north, south, and southeast aspects (with 2286 lakes (51.98%) and 4028.28 ha area (50.00%)). Thermokarst lakes were significantly developed in warm permafrost region with MAGT > − 1.5 ^∘^C, accounting for 47.39% (1879) and 54.38% (2214.69 ha) of the total count and coverage, respectively. Between 1988 and 2020, although small of thermokarst lake shrunk or even had drained, there was a general expansion trend of thermokarst lake with increase in number of 195 (8.58%) and area of 1160.19 ha (41.36%). They decreased by 1404 (61.74%) and 1520.55 ha (54.21%) during 1988–1995 (at rates of − 702 each year and − 706.27 ha/yr) and then increased by 1599 (183.79%) and 2,680.74 ha (208.71%) in number and area of lakes during 1995–2020 (at rates of 184.96 each year and 360.82 ha/yr). Moreover, this significant expansion of thermokarst lakes was attributed to ground ice melting as rising air temperature at a rate of 0.03–0.04 ^∘^C/yr, followed by the increasing precipitation (1.76–3.07 mm/yr) that accelerated the injection of water into the lake.

### Supplementary Information

Supplementary Tables.
