# Supplementary material for: Accelerating thermokarst lake changes on the Qinghai–Tibetan Plateau
Source: Sci Rep. 2024 Feb 5;14:2985. doi: 10.1038/s41598-024-52558-7 (PMC10844240; doi:10.1038/s41598-024-52558-7)
Supplement: Supplementary file 1 — Supplementary Tables. [file 41598_2024_52558_MOESM1_ESM.pdf]

Supporting Information for

Accelerating thermokarst lake changes on the Qinghai-Tibetan Plateau

Guanghao Zhou[1], Wenhui Liu[1]*, Changwei Xie[2], Xianteng Song[3], Qi Zhang[1], Qingpeng Li[1], Guangyue Liu[2], Qing Li[1], Bingnan Luo[1]

1 Department of Geological Engineering, Qinghai University, Xining, Qinghai, 810016, China

2 Cryosphere Research Station on the Qinghai-Tibet Plateau, State Key Laboratory of Cryosphere Sciences, Northwest Institute of Eco-Environment and Resources, Chinese Academy of Sciences, Lanzhou 730000, China

3 Xining Center for Integrated Natural Resources Survey, China Geological Survey, Xining 810000, Qinghai, China

* Corresponding author

Table of Contents

Table S1. Sentinel-2A images for lake extraction

Table S2. Landsat images for long time analysis of lakes

Table S1. Sentinel-2A images for lake extraction

Name
S2A_MSIL2A_20210928T042711_N0301_R133_T46RDV_20210928T074543.SAFE
S2A_MSIL2A_20210928T042711_N0301_R133_T46SCA_20210928T074543.SAFE
S2A_MSIL2A_20210928T042711_N0301_R133_T46SDC_20210928T074543.SAFE
S2A_MSIL2A_20210928T042711_N0301_R133_T46SDD_20210928T074543.SAFE
S2A_MSIL2A_20211008T042711_N0301_R133_T46SCB_20211008T072506.SAFE
S2A_MSIL2A_20211008T042711_N0301_R133_T46SCC_20211008T072506.SAFE
S2B_MSIL2A_20211013T042729_N0301_R133_T46RCU_20211013T064657.SAFE
S2B_MSIL2A_20211013T042729_N0301_R133_T46RCV_20211013T064657.SAFE
S2B_MSIL2A_20211013T042729_N0301_R133_T46SEE_20211013T073505.SAFE

Table S2. Landsat images for long time analysis of lakes

Time	Name
1988	LT05_L1TP_137035_19880914_20200917_02_T1
1988	LT05_L1TP_137036_19880914_20200917_02_T1
1988	LT05_L1TP_137037_19880914_20200917_02_T1
1988	LT05_L1TP_137038_19880914_20200917_02_T1
1988	LT05_L1TP_137039_19880914_20200917_02_T1
1988	LT05_L1TP_138037_19880430_20200917_02_T1
1991	LT05_L1TP_137035_19911009_20201029_02_T1
1991	LT05_L1TP_137036_19911009_20200915_02_T1
1991	LT05_L1TP_137037_19911009_20200915_02_T1
1991	LT05_L1TP_137038_19911009_20200915_02_T1
1991	LT05_L1TP_137039_19911009_20200915_02_T1
1991	LT05_L1TP_138037_19910914_20200915_02_T1
1995	LT05_L1TP_137035_19950817_20200912_02_T1
1995	LT05_L1TP_137036_19950817_20200912_02_T1
1995	LT05_L1TP_137037_19950817_20200912_02_T1
1995	LT05_L1TP_137038_19950817_20200912_02_T1
1995	LT05_L1TP_137039_19950817_20200912_02_T1
1995	LT05_L1TP_138037_19951011_20200912_02_T1
1997	LT05_L1TP_137035_19970822_20200910_02_T1
1997	LT05_L1TP_137036_19970822_20200910_02_T1
1997	LT05_L1TP_137037_19970822_20200910_02_T1
1997	LT05_L1TP_137038_19970822_20200910_02_T1
1997	LT05_L1TP_138037_19970117_20200910_02_T1
1997	LT05_L1TP_138039_19970218_20200911_02_T1
2002	LT05_L1TP_137035_20021007_20200905_02_T1
2002	LT05_L1TP_137036_20021007_20200905_02_T1
2002	LT05_L1TP_137037_20021007_20200905_02_T1
2002	LT05_L1TP_137038_20021007_20200905_02_T1
2003	LT05_L1TP_137039_20031213_20200904_02_T1

2002	LT05_L1TP_138037_20020624_20200905_02_T1
2007	LT05_L1TP_137035_20070919_20200829_02_T1
2007	LT05_L1TP_137036_20070919_20200830_02_T1
2007	LT05_L1TP_137037_20070919_20200830_02_T1
2007	LT05_L1TP_137038_20070802_20200829_02_T1
2007	LT05_L1TP_137039_20070122_20200831_02_T1
2007	LT05_L1TP_138037_20070505_20200830_02_T1
2009	LT05_L1TP_137039_20091026_20200825_02_T1
2009	LT05_L1TP_138036_20090830_20200825_02_T1
2009	LT05_L1TP_138037_20090830_20200825_02_T1
2009	LT05_L1TP_138038_20090830_20200825_02_T1
2009	LT05_L1TP_137035_20100725_20200823_02_T1
2009	LT05_L1TP_137036_20100725_20200823_02_T1
2009	LT05_L1TP_137038_20100725_20200823_02_T1
2011	LT05_L1TP_137035_20110202_20200823_02_T1
2011	LT05_L1TP_137036_20110914_20200820_02_T1
2011	LT05_L1TP_137037_20110914_20200820_02_T1
2011	LT05_L1TP_137038_20110914_20200820_02_T1
2011	LT05_L1TP_137039_20110914_20200820_02_T1
2011	LT05_L1TP_138037_20111007_20200820_02_T1
2013	LC08_L1TP_137035_20130802_20200912_02_T1
2013	LC08_L1TP_137036_20130802_20200912_02_T1
2013	LC08_L1TP_137037_20130802_20200912_02_T1
2013	LC08_L1TP_137038_20130802_20200912_02_T1
2013	LC08_L1TP_138037_20151002_20200908_02_T1
2013	LC08_L1TP_138039_20131012_20200913_02_T1
2015	LC08_L1TP_137035_20150723_20200908_02_T1
2015	LC08_L1TP_137036_20150723_20200908_02_T1
2015	LC08_L1TP_137037_20150723_20200908_02_T1
2015	LC08_L1TP_137038_20150723_20200908_02_T1
2015	LC08_L1TP_138037_20151002_20200908_02_T1
2015	LC08_L1TP_138038_20151002_20200908_02_T1
2015	LC08_L1TP_138039_20151002_20200908_02_T1
2017	LC08_L1TP_137035_20180715_20200831_02_T1
2017	LC08_L1TP_137036_20180715_20200831_02_T1
2017	LC08_L1TP_137037_20170712_20200903_02_T1
2017	LC08_L1TP_137038_20171016_20200902_02_T1
2017	LC08_L1TP_138036_20171007_20200903_02_T1
2017	LC08_L1TP_138037_20171007_20200903_02_T1
2017	LC08_L1TP_138038_20171007_20200903_02_T1
2017	LC08_L1TP_138039_20171023_20200902_02_T1
2020	LC08_L1TP_137035_20201008_20201016_02_T1
2020	LC08_L1TP_137036_20201008_20201016_02_T1

2020	LC08_L1TP_137037_20201008_20201016_02_T1
2020	LC08_L1TP_137038_20201008_20201016_02_T1
2020	LC08_L1TP_137039_20201008_20201016_02_T1
2020	LC08_L1TP_138037_20201015_20201105_02_T1
2020	LC08_L1TP_138038_20201015_20201105_02_T1
